# Familial Risk for Chronic Disease and Intent to Share Family History With a Health Care Provider Among Urban Appalachian Women, Southwestern Ohio, 2007

**Published:** 2009-12-15

**Authors:** Melanie F. Myers, Margaret G. Au, Nancy S. Warren, Sandra J. Cornett, Todd G. Nick, Yu Wang, Jody Wallace

**Affiliations:** Cincinnati Children’s Hospital Medical Center. Dr Myers is also affiliated with the University of Cincinnati, Cincinnati, Ohio; University of Cincinnati and Cincinnati Children’s Hospital Medical Center, Cincinnati, Ohio; University of Cincinnati and Cincinnati Children’s Hospital Medical Center, Cincinnati, Ohio; Ohio State University College of Medicine, Columbus, Ohio; Cincinnati Children’s Hospital Medical Center, Cincinnati, Ohio; Cincinnati Children’s Hospital Medical Center, Cincinnati, Ohio; University of Cincinnati and Cincinnati Children’s Hospital Medical Center, Cincinnati, Ohio, and St Elizabeth Medical Center, Edgewood, Kentucky

## Abstract

**Introduction:**

Family history of certain chronic diseases is a risk factor for those diseases. We assessed demographic characteristics associated with familial risk for common diseases and whether familial risk was associated with intent to share family history with a health care provider among urban Appalachian women.

**Methods:**

Urban Appalachian women (N = 88) with less than a college education participated in education sessions about family history in health promotion in southwest Ohio. Participants used My Family Health Portrait, electronically or on paper, to document their level of familial risk. Evaluations completed after each session gauged intent to share family history with a health care provider.

**Results:**

Participants who used the paper version of My Family Health Portrait had lower odds of high familial risk for diabetes, heart disease, and stroke. Most participants (n = 62, 77%) reported that they intended to share their family history with a health care provider. Factors associated with intent to share family history included younger age, use of the electronic family history tool, and high familial risk of heart disease.

**Conclusion:**

The large proportion of women who intended to share family history with a health care provider may reflect the success of the educational component. Since familial risk for chronic disease is high among these urban Appalachian women, the need to share family history should continue to be promoted.

## Introduction

Many people from rural Appalachia migrated to cities outside Appalachia after World War II, including Cincinnati and Dayton, Ohio (1). The prevalence of many chronic diseases and risk factors is higher in Appalachians than in others in this region (2), so Appalachians would benefit from knowing their familial risk for these diseases and sharing this information with their health care providers. Awareness of familial risk for a disease may motivate people to be screened (3-5). Appalachian women in West Virginia recognized that a family history of cancer is an indication for more frequent cancer screenings (6). In addition, a family history of colon cancer was strongly associated with having had a colonoscopy among Appalachians in the Ohio Valley (7).

Familial risk for heart disease, diabetes, stroke, breast cancer, ovarian cancer, and colon cancer has been described in the general population in the United States (5,8-11) but not in the urban Appalachian population. Also not well studied is the relationship between having a family history of a disease and intent to share this information with a health care provider. Understanding factors associated with familial risk and intent to share family history can inform promotional efforts on the use of family history in urban Appalachian and other communities. The goal of this study was to determine whether demographic characteristics were associated with familial risk and whether familial risk of common diseases predicted intent to share family history with a health care provider among urban Appalachian women in southwestern Ohio.

## Methods

This study was part of the Family History Demonstration Project, details of which have been reported elsewhere (12). Briefly, 6 participating community organizations that serve urban Appalachian populations in Cincinnati, Ohio (2 organizations), Dayton, Ohio (3 organizations), and Newport, Kentucky (1 organization) (northern Kentucky is a part of the Greater Cincinnati metropolitan statistical area), recruited participants. A representative from each organization invited potential participants to attend initial and follow-up educational sessions about the use of family history in health care. The community organizations are nonprofit social service agencies that provide resources such as career training, social services, and child care. None serves only Appalachians, but all are in neighborhoods where many Appalachians live. The Cincinnati Children's Hospital institutional review board deemed the Family History Demonstration Project exempt from review because it was an educational project, not research on human participants.

We recruited women because of their influential role in family health (13) and because women are more likely than men to record their family history (14). One of our goals, reported elsewhere (12), was to create educational tools for urban Appalachians of limited literacy. As a proxy for literacy, a requirement for participation was less than a college education. The Appalachian heritage criterion, which was defined by the community organizations, was met if the participant, a parent, or a grandparent was born in 1 of the 410 counties designated by the Appalachian Regional Commission (15) or if the participant self-identified as Appalachian. From July through October 2007, we held 13 initial and 12 follow-up sessions (2 follow-up sessions were combined) at the 4 community organizations that had computers with Internet access.

Education sessions focused on family history as a risk factor for common diseases and how to collect and document family history by using My Family Health Portrait (16) electronically or on paper (participant's choice). Participants recorded and updated family histories during both education sessions. Between sessions — approximately 2 weeks — participants were asked to speak with family members and update their histories.

After the follow-up sessions, participants completed a survey to assess their intent to share their family history with a health care provider. We also interviewed participants on the telephone (10- to 15-minute interviews) approximately 4 weeks after they attended the last education session. We asked questions such as "Have you talked to a health care provider about your family history since our last meeting?" and "If no, do you intend to talk to a health care provider about your family history in the future?" We made up to 5 attempts to contact participants by telephone.

We used family histories and a general familial risk stratification guideline (Figure) (8) to calculate familial risks for heart disease, diabetes, stroke, breast cancer, ovarian cancer, and colon cancer among 88 participants who met the Appalachian criterion. We classified participants as having high, moderate, and average familial risk. If a participant had a given condition, we calculated familial risk twice: including the affected participant as an additional first-degree relative with the condition (17) and not including the affected participant.

**Figure:**
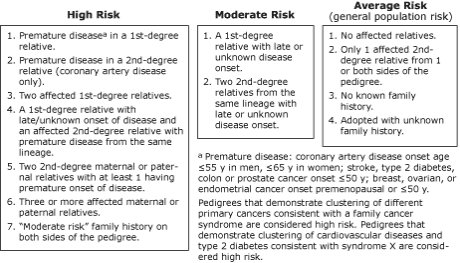
General familial risk stratification guideline. Adapted from Scheuner et al (8). Reprinted with permission of John Wiley & Sons, Inc.

**Table:** 

The figure shows how people are stratified into high, moderate, or average risk categories on the basis of their familial risk for a condition.
A person is considered at high risk if he or she meets any of the following: 1. Premature disease in a first-degree relative. Premature disease is considered coronary artery disease onset age 55 years or younger in men or 65 years or younger in women; stroke, type 2 diabetes, colon or prostate cancer onset 50 years or younger; breast, ovarian, or endometrial cancer onset premenopausal or 50 years or younger. 2. Premature disease in a second-degree relative (coronary artery disease only). 3. Two affected first-degree relatives. 4. A first-degree relative with late/unknown onset of disease and an affected second-degree relative with premature disease from the same lineage. 5. Two second-degree maternal or paternal relatives with at least 1 having premature onset of disease. 6. Three or more affected maternal or paternal relatives. 7. “Moderate risk” family history on both sides of the pedigree.
A person is considered at moderate risk if he or she meets either of the following: 1. A first-degree relative with late or unknown disease onset. 2. Two second-degree relatives from the same lineage with late or unknown disease onset.
A person is considered at average risk (general population risk) if he or she meets any of the following: 1. No affected relatives. 2. Only 1 affected second-degree relative from 1 or both sides of the pedigree. 3. No known family history. 4. Adopted with unknown family history.
Pedigrees that demonstrate clustering of different primary cancers consistent with a family cancer syndrome are considered high risk. Pedigrees that demonstrate clustering of cardiovascular diseases and type 2 diabetes consistent with syndrome X are considered high risk.

After we assessed familial risk for all 6 conditions, breast, ovarian, and colon cancer were omitted from further analyses because of lack of variance in the risk categories (all >80% "average" risk). Correlations between familial risks for heart disease, diabetes, and stroke were calculated by using a Spearman correlation coefficient (*r_s_*).

We used the proportional odds model (18) to conduct unadjusted (crude) and adjusted ordinal logistic regression analyses that used familial risks for heart disease, diabetes, and stroke as outcome variables. Predictor variables included education, age, community organization where the participant attended sessions, family history tool used (electronic or paper), and race. Education was measured as the highest grade completed and collapsed into 3 categories: 6th through 8th grade, 9th through 11th grade, and 12th grade or higher or General Educational Development certification.

We used χ^2^ tests to assess the relationships between familial risk of heart disease, diabetes, and stroke and intent to share family history with a health care provider. Simple and multiple binary logistic regression analyses were carried out to examine predictors of intent to share family history with a health care provider. All statistical modeling was conducted by using the Hmisc and Design packages of S-Plus version 8.0 (Tibico, Inc, Palo Alto, California). Differences were considered significant at *P* ≤ .05.

## Results

A total of 100 women participated in initial education sessions, 92 returned to a follow-up session, and 58 were interviewed over the telephone. Eighty-eight of the 100 participants met the Appalachian criteria, 5 did not know their county of birth but were themselves or had relatives from a state with Appalachian counties, 6 did not meet or were unsure if they met the criteria, and 1 was missing this information (Table 1).

### Familial risk

When affected participants were excluded, 74 of 88 had a high or moderate familial risk for at least 1 of the 6 conditions. Heart disease had the most participants with a high or moderate familial risk, and breast and colon cancer had the fewest (Table 2). Familial risks changed only slightly when affected participants were included in the risk calculations as first-degree relatives. In the following results, familial risks refer to those calculated with affected participants excluded. Forty-nine (56%) participants had high or moderate familial risk for more than 1 condition (31 [35%] were at risk for 2 conditions, 13 [15%] for 3 conditions, 4 [5%] for 4 conditions, and 1 [1%] for 5 conditions).

Familial risk for heart disease was significantly correlated with risk for stroke (*r_s_* = 0.31, *P* = .003) but not with risk for diabetes (*r_s_* = 0.19, *P* = .08). Familial risk for stroke was nonsignificantly correlated with risk for diabetes (*r_s_* = 0.20, *P* = .06).

Familial risk for diabetes and stroke differed significantly according to the family history tool used (diabetes χ^2^ = 7.35, *df* = 2, *P* = .03; stroke χ^2^ = 10.26, *df* = 2, *P* = .006) (Table 3). Previous analyses showed a nonsignificant trend of younger and more educated participants using the electronic tool (12). Participants at the Kentucky location were significantly more likely to use the electronic tool than were participants at other locations (χ^2^ = 6.89, *df* = 2, *P* = .03).

In the adjusted model, older participants had significantly higher odds of high or moderate familial risk for heart disease than did younger participants (Table 4). In both models, participants who used the paper tool had significantly lower odds of high or moderate familial risk of heart disease than did those who used the electronic tool. Type of tool used was the only variable significantly associated with familial risk for diabetes and stroke. Participants who used the paper tool had lower odds of high or moderate familial risk for diabetes and stroke than did those who used the electronic tool.

### Intent to share family history

In the follow-up education session evaluation, 62 (77%) of 81 Appalachian participants indicated that they intended to share their family history with a health care provider. The intent gauged in follow-up sessions was not significantly associated with familial risk for heart disease, diabetes, or stroke. Because the familial risk of heart disease was significantly correlated with stroke (*r_s_* = 0.31, *P* = .003), only familial risks of heart disease and diabetes were included in the multiple logistic regression model (Table 5). In the adjusted model, participants who used the electronic tool were 7 times as likely as those who used the paper tool to intend to share their family history with a health care provider.

Younger participants and those at high risk for heart disease were also more likely to intend to share their family history, although the association with heart disease risk was significant only in the adjusted model (Table 5). Stroke risk was not significantly related to intent to share family history, and including stroke risk in the multiple logistic regression did not change the significance of the other covariates (data not shown).

Telephone interviews offered insight into intent to share family history with a health care provider. Of the 58 participants we reached by phone, 53 met the Appalachian criteria. During the telephone interviews, 20 (38%) said that they had talked to a health care provider about their family history since they participated in the Family History Demonstration Project. Of the 33 participants who had not spoken with a health care provider, 32 reported they intended to do so; most of these 32 explained that they had not spoken with a provider because they were busy or had not yet had a reason to see a provider, and 1 participant cited financial concerns.

We asked the 20 participants who had talked with their provider about their family history to describe the experience. The stated benefits of their experiences included a discussion of the participant's risk factors, increased screening recommendations, and suggested behavioral changes. Participants who had yet to speak with their providers indicated that their reasons for intending to do so included wanting to learn about their risk factors, improving their health, and collecting the information for their children's sake.

## Discussion

High familial risk for heart disease and younger age predicted intent to share family history with a health care provider among urban Appalachian women in southwestern Ohio. Previous research has shown that familial risk for heart disease increased the likelihood of adhering to an aspirin regimen and obtaining cholesterol screening but not of implementing dietary changes, exercising, or quitting smoking (4). Younger participants are less at risk for chronic disease, so their willingness to share family history may be because they perceive more of a disease prevention benefit or because of other generational differences.

Familial risk for diabetes was not associated with intent to share family history, possibly because more participants had diabetes (n = 13) than heart disease (n = 4). Participants who already had diabetes may not have seen any benefit to sharing family history. Alternatively, having a disease increases the likelihood that a person would be under a physician's care, which would increase the number of opportunities for sharing family history.

In general, intent to share family history was high, which suggests that the education sessions were successful in conveying the message that sharing family history with a health care provider can be an effective tool in disease prevention and early diagnosis. The high familial risk of chronic disease in this population supports the continued promotion of the need to share family history.

The type of tool used was associated with familial risk and intent to share family history. In a previous study, Web-based interventions increased health knowledge and improved health behaviors compared with non–Web-based interventions (19). My Family Health Portrait does not make health recommendations, but completing it electronically could have increased participants' confidence in the results. The association between type of tool used and familial risk might reflect characteristics of the tool. When using the electronic tool, participants were prompted for each of the 6 conditions, so they did not have to remember them or worry about spelling errors. The paper tool had only blank lines and no prompts. The lack of prompts in the paper tool may be partially responsible for the lower familial risk in participants who chose the paper tool. We did not assess reasons for choosing the electronic or paper tool.

The prevalences of familial risks for heart disease, diabetes, and ovarian cancer were higher in our sample than in others (8). These higher prevalences could reflect economic, educational, behavioral, or cultural health disparities. The prevalences of familial risks for stroke, breast cancer, and colon cancer were lower in our sample than in others (8,9,11), which may be explained by the lack of specificity in documentation for cancer. In 50 cases, an unspecified "cancer" was documented on participants' family history. Cancer type and age at diagnosis may have less validity as a familial health risk in more distant relatives (20), although this is not well studied for other common diseases (9). In addition, we included both male and female relatives in the denominators when calculating familial risk for breast cancer, and other studies may include only women.

Significant correlations between familial risks for heart disease, diabetes, and stroke have been reported previously (9). Additional study of the risks for 1 disease, such as stroke, when a patient has a family history of another disease, such as diabetes, could guide risk assessment and screening. If health care providers note an increased familial risk for 1 disease, then perhaps they may be prompted to screen for other diseases.

### Limitations

Intent to share family history with a health care provider was common and could reflect the success of the education sessions. In the absence of a control population, we cannot determine whether the same factors would be associated with intent among Appalachian women who had not undergone the family history education.

We did not assess participants' knowledge of diseases or understanding of their own familial risk. Perception of risk and knowledge of disease could affect intent to share family history and should be explored further. We also did not review medical records or death certificates to assess validity of collected family histories and potential misclassification. Therefore, whether the disease prevalence reported is more accurate in the electronic or paper version of My Family Health Portrait cannot be determined.

Since the end of the Family History Demonstration Project education sessions in October 2007, the format of the paper and electronic versions of My Family Health Portrait has changed. At the time of our study, the paper tool resembled a pedigree (pictorial representation of familial relationships), and the current paper tool consists of several tables. The electronic tool now contains a menu of health conditions and is no longer limited to the 6 conditions originally assessed in this study. Our analyses reflect the older formats and should be compared with studies that use the newer formats to determine whether the structure of the tool influences reported familial risk.

Social desirability bias could be another limitation in reporting intent to share family history. This bias has been reported for cancer screening (21), although asking about intent first may produce more honest responses (22). In the telephone interviews, we elicited intent only after participants stated that they had not yet shared their family history with a health care provider.

Our analyses were limited to 88 participants. The small sample size and focused study on urban Appalachians who live in cities outside Appalachia limits the generalizability of results to other populations. However, our findings may be applicable to other communities with similar education characteristics.

### Implications and future directions

Women in this urban Appalachian population are at increased familial risk for common conditions and could benefit from sharing their family history with their health care providers. As people move out of Appalachia and lose cultural ties with the region, it will be interesting to note whether risk factors change and familial risk decreases. In our sample, intent to share family history with a health care provider was common, which could reflect the success of the education sessions. These types of interventions could promote knowledge of family history as a risk factor for disease and encourage action to maintain or improve health.

## Figures and Tables

**Table 1:** Characteristics of Women Who Attended an Initial Educational Session on Family Health History, Southwestern Ohio, 2007

**Characteristic**	All Participants, n (%) (N = 100)^a^	Appalachian Participants, n (%) (N = 88)^a^
**Race**		
White^b^	78 (78)	74 (84)
Black^c^	18 (18)	12 (14)
"White/black"	2 (2)	2 (2)
**Age, y**		
19-29	36 (36)	33 (38)
30-39	22 (22)	18 (21)
40-49	16 (16)	16 (18)
50-59	16 (16)	14 (16)
≥60	9 (9)	7 (8)
**Education**		
6th-8th grade	17 (17)	15 (17)
9th-11th grade	33 (33)	28 (32)
≥12th grade or GED	49 (49)	45 (51)
**Participating community organization**		
Cincinnati, Ohio	43 (43)	40 (46)
Dayton, Ohio	25 (25)	17 (19)
Newport, Kentucky	32 (32)	31 (35)
**Family health history tool used**		
Electronic	51 (51)	43 (49)
Paper	49 (49)	45 (51)

Abbreviation: GED, General Educational Development test.

a Percentages may not total 100 because of missing data and rounding.

b Included participants who identified as "white/Cherokee."

c Included participants who identified as "black/Cherokee."

**Table 2:** Familial Risk for 6 Conditions According to the General Familial Risk Stratification Guideline Among 88 Urban Appalachian Women, Southwestern Ohio, 2007

Condition	Risk	Affected Participants Included, n (%)^a^	Affected Participants Excluded, n (%)^a^
**Heart disease**	Average	26 (30)	26 (30)
	Moderate	13 (15)	14 (16)
	High	49 (56)	48 (55)
**Diabetes**	Average	46 (52)	47 (53)
	Moderate	7 (8)	8 (9)
	High	35 (40)	33 (38)
**Stroke**	Average	64 (73)	65 (74)
	Moderate	11 (13)	12 (14)
	High	13 (15)	11 (13)
**Breast cancer**	Average	80 (91)	80 (91)
	Moderate	4 (5)	4 (5)
	High	4 (5)	4 (5)
**Ovarian cancer**	Average	75 (85)	77 (88)
	Moderate	5 (6)	5 (6)
	High	8 (9)	6 (7)
**Colon cancer**	Average	86 (98)	86 (98)
	Moderate	1 (1)	1 (1)
	High	1 (1)	1 (1)

a Percentages may not total 100 because of rounding.

**Table 3:** Familial Risk for 3 Conditions by Type of Family History Tool Used to Assess Risk Among 88 Urban Appalachian Women, Southwestern Ohio, 2007

Disease	Risk	Paper Tool, n (%)(n = 45)^a^	Electronic Tool, n (%)(n = 43)^a^	*P* Value
**Heart disease **	Average	16 (36)	10 (23)	.15
	Moderate	9 (20)	5 (12)	
	High	20 (44)	28 (65)	
**Diabetes **	Average	28 (62)	19 (44)	.03
	Moderate	6 (13)	2 (5)	
	High	11 (24)	22 (51)	
**Stroke **	Average	39 (87)	26 (61)	.006
	Moderate	5 (11)	7 (16)	
	High	1 (2)	10 (23)	

a Percentages may not total 100 because of rounding.

**Table 4:** Predictors of High or Moderate Familial Risk^a^ for 3 Conditions Among 85^b^ Urban Appalachian Women, Southwestern Ohio, 2007

Condition	Predictor	Crude OR (95% CI)	*P* Value	Adjusted OR (95% CI)	*P* Value
**Heart disease**	**Age, y**				
	26 (25th percentile)	1 [Reference]	.06	1 [Reference]	.04
	47 (75th percentile)	1.91 (0.98-3.71)		2.16 (1.03-4.55)	
	**Education, highest grade completed**				
	6th-8th	1.85 (0.52-6.54)	.61	1.60 (0.42-6.12)	.79
	9th-11th	1 [Reference]		1 [Reference]	
	≥12th or GED	1.08 (0.44-2.69)		1.15 (0.41-3.23)	
	**Tool used**				
	Electronic	1 [Reference]	.05	1 [Reference]	.009
	Paper	0.43 (0.18-1.00)		0.28 (0.11-0.73)	
	**Location**				
	Cincinnati	1 [Reference]	.61	1 [Reference]	.65
	Dayton	0.64 (0.22-1.86)		0.56 (0.15-1.99)	
	Newport	0.67 (0.26-1.73)		0.71 (0.21-2.36)	
	**Race**				
	Black	1 [Reference]	.13	1 [Reference]	.32
	White	2.40 (0.78-7.43)		1.92 (0.54-6.86)	
**Diabetes**	**Age, y**				
	26 (25th percentile)	1 [Reference]	.38	1 [Reference]	.26
	47 (75th percentile)	1.32 (0.71-2.46)		1.51 (0.74-3.04)	
	**Education, highest grade completed**				
	6th-8th	1.06 (0.30-3.76)	.71	1.12 (0.28-4.46)	.70
	9th-11th	1 [Reference]		1 [Reference]	
	≥12th or GED	1.44 (0.57-3.64)		1.54 (0.55-4.30)	
	**Tool used**				
	Electronic	1 [Reference]	.02	1 [Reference]	.01
	Paper	0.37 (0.16-0.86)		0.29 (0.11-0.76)	
	**Location**				
	Cincinnati	1 [Reference]	.73	1 [Reference]	.49
	Dayton	1.44 (0.49-4.20)		0.87 (0.25-2.97)	
	Newport	0.92 (0.35-2.38)		0.49 (0.15-1.64)	
	**Race**				
	Black	1 [Reference]	.21	1 [Reference]	.19
	White	0.48 (0.15-1.51)		0.41 (0.11-1.55)	
**Stroke**	**Age, y**				
	26 (25th percentile)	1 [Reference]	.59	1 [Reference]	.37
	47 (75th percentile)	1.22 (0.60-2.48)		1.47 (0.63-3.44)	
	**Education, highest grade completed**				
	6th-8th	0.79 (0.20-3.18)	.93	0.71 (0.16-3.25)	.81
	9th-11th	1 [Reference]		1 [Reference]	
	≥12th or GED	0.83 (0.29-2.40)		0.70 (0.21-2.29)	
	**Tool used**				
	Electronic	1 [Reference]	.004	1 [Reference]	.003
	Paper	0.22 (0.08-0.62)		0.18 (0.06-0.56)	
	**Location**				
	Cincinnati	1 [Reference]	.98	1 [Reference]	.89
	Dayton	0.90 (0.24-3.33)		0.76 (0.17-3.29)	
	Newport	1.02 (0.36-2.94)		0.75 (0.20-2.84)	
	**Race**				
	Black	1 [Reference]	.78	1 [Reference]	.89
	White	1.05 (0.26-4.24)		1.12 (0.23-5.43)	

Abbreviations: OR, odds ratio; CI, confidence interval; GED, General Educational Development test.

a Risk calculated with affected participants excluded.

b Three participants were not included in the model because they lacked information about race (1 with missing data, 2 categorized as "white/black").

**Table 5:** Predictors of Intent to Share Family History With a Health Care Provider Among 78^a^ Urban Appalachian Women, Southwestern Ohio, 2007

Predictor^b^	Crude OR (95% CI)	*P* Value	Adjusted OR (95% CI)	*P* Value
**Age, y**				
26 (25th percentile)	1 [Reference]	.04	1 [Reference]	.01
47 (75th percentile)	0.42 (0.19-0.96)		0.14 (0.03-0.62)	
**Education, highest grade completed**				
6th-8th	1.43 (0.30-6.70)	.64	5.57 (0.47-65.45)	.35
9th-11th	1 [Reference]		1 [Reference]	
≥12th or GED	1.78 (0.54-5.88)		0.99 (0.11-9.26)	
**Tool used**				
Electronic	1 [Reference]	.008	1 [Reference]	.04
Paper	0.16 (0.04-0.61)		0.14 (0.02-0.92)	
**Location**				
Cincinnati	1 [Reference]	.52	1 [Reference]	.42
Dayton	2.41 (0.46-12.61)		2.40 (0.15-37.5)	
Newport	1.56 (0.46-5.25)		0.38 (0.04-3.76)	
**Heart disease risk**				
Average	1 [Reference]	.11	1 [Reference]	.02
Moderate	1.46 (0.33-6.46)		3.89 (0.38-40.11)	
High	3.79 (1.07-13.47)		33.71 (2.73-416.58)	
**Diabetes risk**				
Average	1 [Reference]	.92	1 [Reference]	.37
Moderate	1.52 (0.16-14.53)		2.20 (0.10-49.42)	
High	1.16 (0.37-3.65)		0.34 (0.06-1.93)	

Abbreviations: OR, odds ratio; CI, confidence interval; GED, General Educational Development test.

a Seven participants were not included in the model because of missing data on intent to share family history and 3 because they lacked information about race (1 with missing data, 2 categorized as "white/black").

b Race was not included in these results because all black respondents (n = 11) said that they intended to share family history with a health care provider. The resulting nonsignificant ORs were zero with wide CIs.
